# BOPPPS model with virtual simulation system for otorhinolaryngology head and neck surgery nursing interns: a quasi-experimental study

**DOI:** 10.1186/s12909-026-09648-z

**Published:** 2026-06-08

**Authors:** Qi-ling Shen, Yi-mei Gu, Biao-xin Zhang, Li-hua Zhou, Kai-le Wu, Bu-sheng Tong, Ju-fang Yang, Jia-xin Li, Shao-Hua Hu

**Affiliations:** 1 Department of Otolaryngology-Head and Neck Surgery, the First Affiliated Hospital of Anhui Medical University, Hefei, 230022 China; 2 Department of Emergency, the First Affiliated Hospital of Anhui Medical University, Hefei, 230022 China; 3 School of Nursing, Anhui Medical University, Hefei, 230032 China; 4 The Nursing Department, the First Affiliated Hospital of Anhui Medical University, Hefei, 230022 China

**Keywords:** BOPPPS model, Virtual simulation system, Otolaryngology and head and neck surgery, Nursing interns

## Abstract

**Background:**

Clinical training in surgical nursing faces limitations in safety and consistency. A virtual simulation system addresses safety but lacks pedagogical structure. Integrating it with the bridge-in, objective, pre-assessment, participatory learning, post-assessment, and summary (BOPPPS) model may enhance learning outcomes for surgical nursing interns.

**Methods:**

This study employed a mixed-methods approach combining a quasi-experimental design with qualitative research. It was conducted among 120 nursing interns, who were assigned to either an intervention group (*n* = 60) or a control group (*n* = 60). The control group received traditional teaching methods, while the intervention group received the BOPPPS model with virtual simulation system. Outcome assessors and data analysts were blinded to group assignment to minimize bias. After the rotation, the two groups were compared in terms of objective learning outcomes including internship performance (theoretical, operational, and clinical practice scores), self-directed learning ability, and humanistic care ability; in addition, teaching satisfaction was also assessed. SPSS 25.0 was used for statistical analysis. Qualitative data were collected through semi-structured interviews and analyzed using thematic analysis.

**Results:**

Baseline comparisons showed no significant differences between the two groups in age, gender, etc. (*P* > 0.05 for all). After the intervention, the intervention group showed significantly higher internship performance than the control group (total scores: 94.86 ± 1.28 vs. 90.29 ± 1.62, *P* < 0.05, d = 3.12, 95% CI: 2.53 to 3.71). The intervention group also showed superior self-directed learning ability (total scores: 106.35 ± 16.08 vs. 99.57 ± 19.93, *P* = 0.043, d = 0.37, 95% CI: 0.01 to 0.73) and higher humanistic care competency (total scores: 203.08 ± 27.52 vs. 187.92 ± 38.71, *P* = 0.013, d = 0.46, 95% CI: 0.09 to 0.82). In addition, teaching satisfaction was significantly higher in the intervention group (98.3% vs. 86.7%; χ² = 6.54, *P* = 0.038). Qualitative analysis of the new teaching model conducted with 14 nursing interns identified five key themes: (1) stimulating learning interest; (2) enhancing self-directed learning ability; (3) improving expressive skills and humanistic care capabilities; (4) strengthening clinical thinking and mastery of nursing skills; (5) suggestions for improving this teaching model.

**Conclusion:**

Beyond improving teaching satisfaction, the BOPPPS model with virtual simulation system was associated with superior improvements in objective learning outcomes such as theoretical knowledge, practical skills, self-directed learning ability, and humanistic care competency among otorhinolaryngology head and neck surgery nursing interns.

**Supplementary Information:**

The online version contains supplementary material available at 10.1186/s12909-026-09648-z.

## Introduction

Otorhinolaryngology Head and Neck Surgery is a specialized discipline focusing on diseases of the ear, nose, throat, and head and neck regions. The anatomical characteristics of narrow passages and deep cavities in this field demand that medical professionals possess strong expertise and clinical skills. As successors in the nursing profession, nursing students require training that emphasizes not only the acquisition of professional knowledge but also the comprehensive development of their overall competencies [1]. Clinical practicum serves as a critical component in achieving this goal. Its purpose is to help students deepen and consolidate foundational knowledge, systematically cultivate clinical thinking skills, and enable them to flexibly apply theoretical knowledge to address specific problems encountered in clinical practice [2]. The current teaching model is predominantly teacher-centered, with students merely participating in class. The lack of effective interaction between teachers and students in teaching activities hinders the development of students’ teamwork skills and critical thinking, thereby affecting their classroom satisfaction [3]. Due to the relative complexity of ear, nose, throat, head, and neck anatomy, students generally find the teaching content overly abstract and difficult to understand, leading to feelings of apprehension. This, in turn, diminishes their enthusiasm for independent learning, reduces self-directed learning behaviors, and results in a decline in learning ability and self-efficacy [4]. In the teaching and assessment of operational skills, there is currently a tendency to emphasize technical training while neglecting the cultivation of humanistic qualities. This leads some nursing students to focus excessively on technical procedures in clinical practice, failing to integrate a sense of care into the nursing process. As a result, the effective implementation of humanistic care in clinical settings is hindered, and the difficulty for nursing students in both their internships and their future work in surgical nursing is increased [5, 6]. Therefore, there is an urgent need to explore new teaching models that can effectively enhance students’ comprehensive clinical competencies [7].

The BOPPPS model comprises six components: Bridge-in, Objective, Pre-assessment, Participatory Learning, Post-assessment, and Summary. It not only emphasizes students’ comprehensive participatory learning in teaching but also provides a holistic, coherent teaching process and theoretical foundation for achieving teaching objectives [8]. The BOPPPS model is a pedagogical approach that emphasizes student engagement and timely feedback, aiming to systematically enhance teaching effectiveness [9]. Research [10] has shown that the BOPPPS model not only significantly improves students’ clinical skills and theoretical knowledge but also enhances their academic performance and learning satisfaction, demonstrating more pronounced effectiveness compared to traditional teaching methods. Currently, the BOPPPS model has been successfully applied in teaching practices across multiple disciplines, including medical education and ophthalmology [11, 12], with positive feedback reported. However, its application in the specific professional field of otorhinolaryngology head and neck surgery has not yet been systematically explored or researched.

A virtual simulation system breaks the constraints of time, space, and the number of operational repetitions [13]. It can simulate a series of patient responses during procedures, allowing students to interact with patients as in real-life scenarios. This not only provides students with a more vivid and intuitive learning experience but also fosters the comprehensive development of their critical thinking, knowledge application, and practical innovation skills [14]. These advantages align closely with the goal of cultivating practical and innovative talents in the new era [15], and also meet the teaching and training objectives of otorhinolaryngology head and neck surgery. Currently, this technology has been successfully applied in various fields such as medical education and obstetrics and gynecology [16, 17], achieving positive outcomes.

Based on these fundings, this study applies the BOPPPS model with virtual simulation system to internship nursing students in otorhinolaryngology head and neck surgery. It aims to construct an integrated smart classroom teaching model that combines online, offline, and clinical practice. This initiative seeks to provide new insights and practical references for clinical teaching reform in otorhinolaryngology head and neck surgery, with the goal of enhancing students’ clinical skills and comprehensive competencies, and promoting the overall improvement of medical education quality.

## Methods

This study used a concurrent embedded mixed-methods design. The quantitative component (quasi-experimental, *n* = 120) served as the primary method, comparing outcomes between the intervention and control groups. The qualitative component (semi-structured interviews, *n* = 14) was concurrently embedded to explore students‘ experiences and help interpret the quantitative findings.

### Participants

Using purposive sampling, the undergraduate nursing interns in our department from April 2023 to April 2025 were selected as the study subjects. Based on the internship period, nursing studentswho completed their 4-week rotation between April 2023 and September 2023 were assigned to the control group (*n* = 60), and those whorotated between October 2024 and April 2025 were assigned to the intervention group (*n* = 60).

The inclusion criteria were: (1) Full-time undergraduate nursing students. (2) No prior experience with BOPPPS model or virtual simulation system in surgical nursing education. (3) Currently free from physical or psychiatric symptoms. (4) Voluntary participation in this study with signed informed consent. The exclusion criteria were: (1) Inability to complete the internship due to reasons such as withdrawal or suspension from studies. (2) Failure to participate in the theoretical or operational assessments. All students had completed the same version of the otorhinolaryngology head and neck surgery nursing course prior to their clinical internship. Demographic and baseline characteristics, including age, gender, pre-course average scores, and learning habits, were assessed before the intervention. The results indicated that the two groups were comparable (*P* > 0.05), as detailed in Table 1.

**Table 1:** Comparison of general information of two groups of nursing students

Item	Intervention group (*n* = 60)	Control group (*n* = 60)	χ^2^/t	*P*
Age	21.00 ± 1.69	21.22 ± 1.25	0.627	0.799
Gender			0.391	0.532
Male	14	17		
Female	46	43		
Place of Origin			0.034	0.853
City	34	35		
Rural area	26	25		
Whether served as a class leader			1.250	0.264
Yes	21	27		
No	39	33		
Three-year major GPA			2.391	0.303
< 70	2	1		
70–80	20	28		
> 80	38	31		
Pre-class preview & post-class review			1.109	0.574
Frequently	33	31		
Occasionally	26	26		
Never	1	3		
Discuss study topics during breaks			2.407	0.300
Frequently	34	28		
Occasionally	26	31		
Never	0	1		

### Teaching methods

Both groups were taught by the same teaching team (2 supervisors and 4 instructors). A single-blind design was adopted: the instructors were aware of group allocation, while outcome assessors and the data analyst were blinded to group assignment to minimize bias.

#### Control group

The traditional teaching method was adopted, wherein the chief supervisor conducted unified in-person group theoretical lectures every Monday, and clinical practice followed a one-on-one mentoring approach. During the first week of internship, nursing students learned about common diseases in the otorhinolaryngology-head and neck surgery department and the nursing knowledge related to various drainage tubes. In weeks 2 to 3, clinical instructors provided theoretical lectures and taught perioperative nursing skills. In week 4, students were guided through nursing ward rounds and participated in the final departmental assessment. The virtual simulation system was unavailable during the control period; thus, control group students received only conventional teaching and had no access to the BOPPPS model with virtual simulation system.

#### Intervention group

#####  Teaching preparation

The teaching team received training on smart classroom instruction, covering the use of Rain Classroom and the virtual simulation system, as well as the BOPPPS model.

##### Teaching implementation

**Fig. 1:**
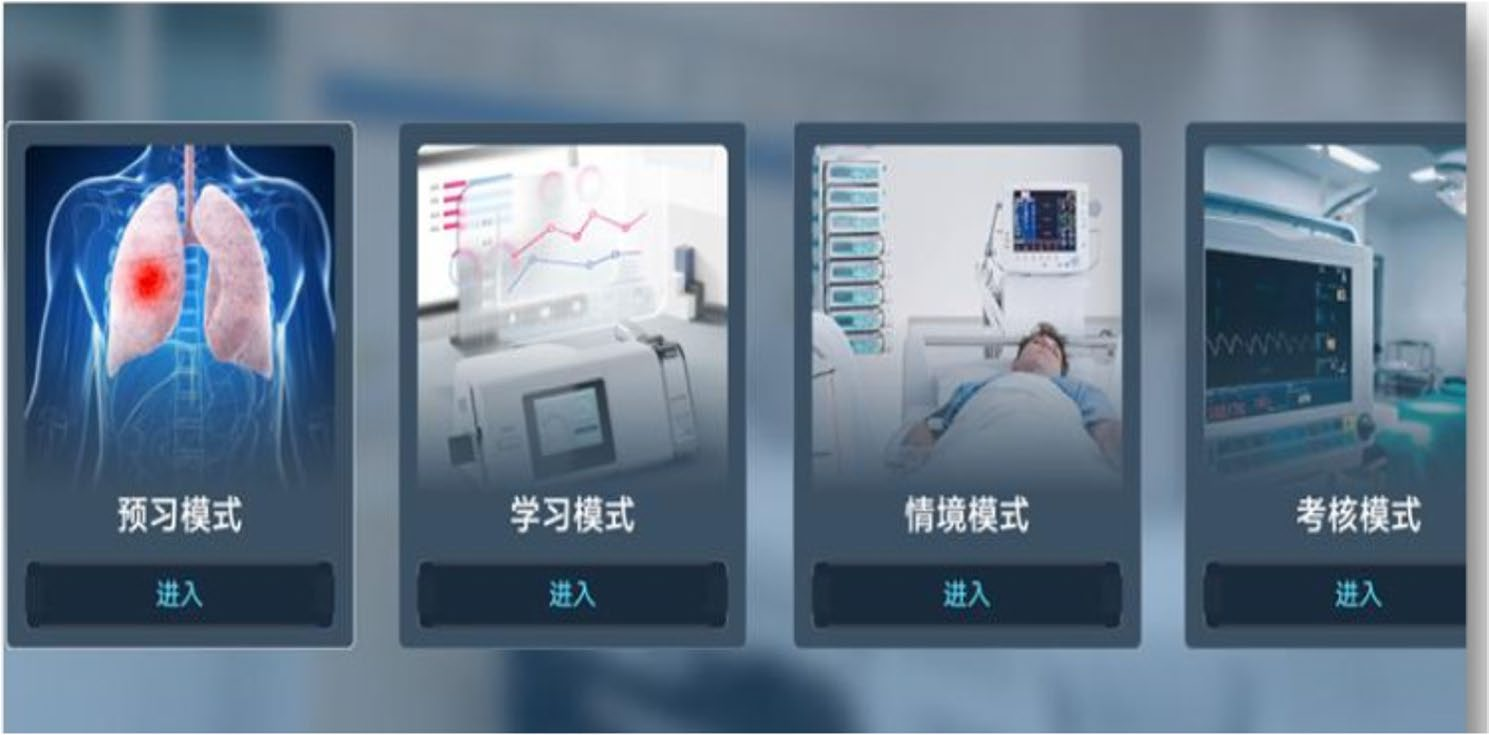
The virtual simulation-based instructional system

**Fig. 2:**
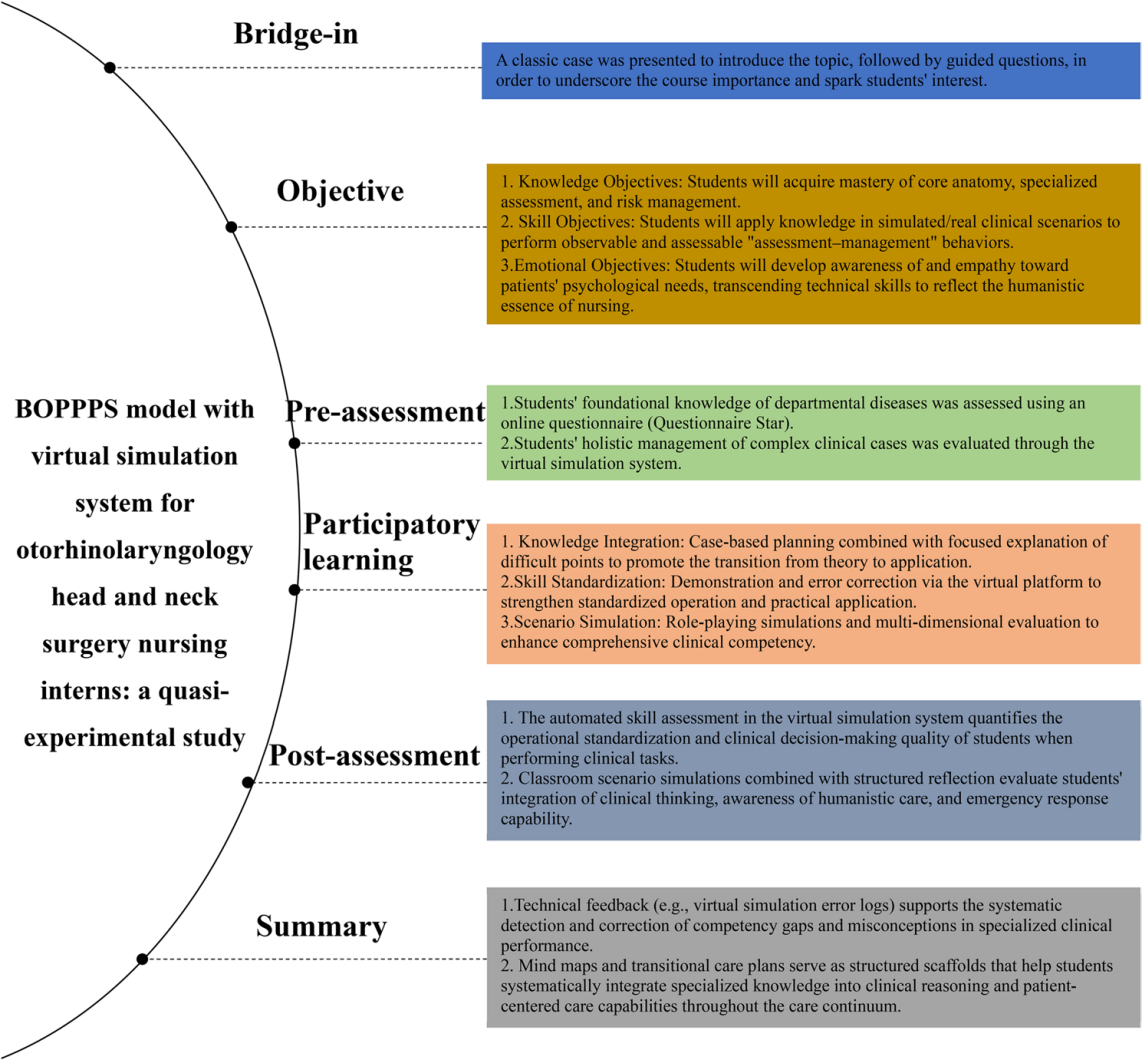
Flowchart of the study design

In addition to the base instruction received by the control group, the intervention group participated in the BOPPPS model with virtual simulation system. The overall instruction followed the six stages of the BOPPPS model, distributed across pre-class, in-class, and post-class activities. The virtual simulation system was facilitated through the National Virtual Simulation Experiment Teaching Course Sharing Platform (abbreviation: virtual simulation system), using the course “Virtual course on endotracheal suctioning for adult invasive mechanical ventilation” (Tongji University) (Fig. 1). The system is high fidelity (VR headsets + haptic feedback; HTC Vive or touchscreens). Fidelity checks confirmed that all four sessions were delivered as planned, with instructor adherence monitored by the chief supervisor (LJX and SQL). Six instructors received 8 h of training on the BOPPPS model and virtual simulation system facilitation before the intervention. The intervention spanned 4 weeks (1 session/week, 60 min/session). Figure 2 demonstrates the clinical teaching design of the BOPPPS model with virtual simulation system.

The instructional design is primarily divided into the following six components:

- Bridge-in (B) Taking the perioperative nursing care for laryngeal cancer patients as an example, play a video of a patient after laryngeal cancer surgery. The content shows the patient after a total laryngectomy, with a tracheostomy tube in the neck, attempting to communicate through gestures, appearing very anxious, and showing a small amount of bright red blood in the neck drainage tube. Then, introduce the questions: Classmates, the patient is unable to speak right now. What is he trying to express? Is the neck bleeding normal? As the responsible nurse, what should your first step be? Guide the nursing interns in responding, and this session will last approximately 5 min.
- Objective (O) The teacher incorporates a virtual case study and clearly outlines three core learning objectives for this lesson: (1) Knowledge Objective: To accurately recite the key observation points for complications such as postoperative bleeding, infection, and breathing difficulties in laryngeal cancer patients. (2) Skill Objective: To carry out suctioning operations for laryngeal cancer patients involving obstructive asphyxia caused by sputum in the virtual simulation system. (3) Emotional Objective: To master effective communication skills with non-verbal patients (e.g., using a communication board) through virtual interaction, and this session will last approximately 3 min.
- Pre-assessment (P) The teachers distributes questions via Questionnaire Star (https://www.wjx.cn/) to gauge students' basic understanding. Randomly select one student to come forward and practice suctioning for the patient on the virtual simulation system. Observe whether the student knows to adjust the negative pressure first and whether to check the suction catheter. The system will immediately display outcomes such as patient mucosal injury or infection risk in response to operational errors (e.g., rough handling, failure to maintain sterile technique), allowing students to visually see the consequences of their mistakes, and this session will last approximately 7 minutes.
- Participation (P) Students, in groups of 4 to 6, enter the virtual training room. Wearing VR headsets or operating touchscreens, they perform tracheostomy care. In the virtual scenario, students role-play as nurses and communicate with the patient. They can use tools such as communication boards or writing boards to comfort the patient and address their needs (e.g., pain, thirst). The system suddenly triggers a critical value alert: the virtual patient experiences continuous neck bleeding and a drop in oxygen saturation (Sp02). The team must collaborate: one person notifies the doctor, another applies pressure to stop the bleeding, and another prepares emergency supplies. The system records the team's coordination and the success rate of the rescue in real time, and this session will last approximately 30 minutes.
- Post-assessment (P) The system automatically generates a scoring report (including operation standardization, complication identification accuracy, and humanistic care score). Two head nurses who did not participate in the teaching will grade based on the surveillance footage, and this session will last approximately 10 minutes.
- Summary (S) The teacher presents the class's high-frequency errors from the virtual operations. Combined with the virtual case, a mind map for perioperative care of laryngeal cancer is outlined (airway management as the core, nutritional support as the foundation, and psychological care as the key). Students are assigned homework: they are required to develop a home care plan for the patient after discharge (including wound self-care and speech rehabilitation training), and this session will last approximately 5 minutes.

### Teaching effectiveness evaluation

Quantitative data included students’ internship performance, self-directed learning ability, humanistic care competence, and teaching satisfaction. In addition, semi-structured interviews were used to collect qualitative data to supplement the evaluation of teaching effectiveness. The questionnaires were administered before the instruction and on the last day of the internship (after the instruction), and all were effectively retrieved.

#### Internship performance

Theoretical and operational assessments were conducted in the final week of the internship. The theoretical assessment was a closed-book examination with a maximum score of 100 points. The exam content was uniformly designed by the teaching team. The operational assessment focused on suctioning via tracheostomy, also with a maximum score of 100 points. The clinical performance score was comprehensively evaluated by the teaching team and supervising instructors during the internship, with a maximum score of 100 points, and the average score was taken. The final internship performance was calculated as follows: clinical performance score (20%) + operational score (40%) + theoretical score (40%).

#### Self-directed learning ability

The Self-Directed Learning Ability Scale (SLAS) for Nursing Undergraduates was employed to evaluate this competency [18]. This instrument comprises three dimensions: Self-Management Ability (10 items), Information Acquisition Ability (11 items), and Learning Cooperation Ability (7 items), totaling 28 items. A 5-point Likert scale was used for scoring, ranging from 1 (completely inconsistent) to 5 (completely consistent). The total score ranges from 28 to 140, with a higher score indicating stronger self-directed learning ability. In this study, the scale demonstrated high internal consistency, with a Cronbach’s α coefficient of 0.941.

#### Humanistic care ability

The Caring Ability Inventory (CAI), translated and culturally adapted by Xu Juan, was used to assess this competency [19]. The scale consists of 37 items across three dimensions: Cognition (14 items), Patience (10 items), and Courage (13 items). Each item is rated on a 7-point Likert scale, ranging from 1 (strongly disagree) to 7 (strongly agree), with some items being reverse-scored. The total score ranges from 37 to 259, and a higher score indicates a stronger caring ability. In this study, the scale demonstrated excellent internal consistency, with a Cronbach’s α coefficient of 0.948.

#### Teaching satisfaction

A self-developed unidimensional scale was used. The response options were: Very Satisfied, Moderately Satisfied, and Dissatisfied. The category Dissatisfied captured any response that was not positive (i.e., including neutral or negative perceptions). The satisfaction rate was calculated as the proportion of students selecting either Very Satisfied or Moderately Satisfied. In this study, the scale demonstrated acceptable internal consistency, with a Cronbach’s α coefficient of 0.870.

To minimize social desirability bias and ensure anonymity, the survey was administered anonymously. Students were informed that their responses would not affect their internship grades or any academic evaluation. Questionnaires were collected by a research assistant who was not involved in teaching or assessment, and no identifying information was recorded. Students were assured that only aggregated data would be reported.

#### Qualitative evaluation – semi-structured interviews

Semi-structured interviews were conducted with 14 nursing students from the intervention group in a hospital classroom setting, who were selected through purposive sampling to ensure diversity in gender, age, and academic performance. Data saturation was achieved after the 12th interview, and two additional interviews were conducted to confirm theme stability. The interview guide included three core questions: (1) What are your thoughts on the application of the BOPPPS model with virtual simulation system in otorhinolaryngology-head and neck surgery? (2) How do you perceive changes in your learning outcomes, clinical hands-on skills, interpersonal communication, and other areas compared to before? (3) What suggestions do you have for the BOPPPS model with virtual simulation system? (Detailed information is provided in Supplementary File S2). Through directed content analysis (a deductive approach), two independent coders conducted the analysis (Cohen’s κ = 0.82). Peer debriefing, audit trails, and the use of anonymous direct quotations for each theme were employed to ensure research rigor. NVivo 14 (QSR International, Melbourne, Australia) was used to facilitate coding.

### Statistical analysis

All data were analyzed using SPSS 25.0 (IBM Corp., Armonk, NY, USA). Measurement data are presented as mean ± standard deviation (SD), with comparisons between groups conducted using the independent samples *t*-test. Count data are expressed as frequency and percentage (%), with intergroup comparisons performed using the Chi-square test. Ordinal data were compared using the Wilcoxon rank-sum test. The significance level was set at *α* = 0.05. Qualitative data obtained from semi-structured interviews were analyzed using thematic analysis, involving content extraction, coding, integration, and the formation of meaningful themes.

### Ethics statement and informed consent

This study was conducted in accordance with the principles of the Declaration of Helsinki and received approval from the Institutional Review Board of Anhui Medical University (approval number: 82240145). Subsequently, permission to carry out the study at the First Affiliated Hospital of Anhui Medical University was granted by its senior administration. All participants were fully informed of the study’s purpose, procedures, and voluntary nature, and each provided written informed consent. Participants were assured that non-participation or withdrawal from the study would not affect their academic evaluations or employment status. Six instructors did not participate in the final scoring, and participants were anonymized in the interviews using codes (S1 to S14). No identifying information was recorded.

### Clinical trial number

not applicable.

### Reporting Standards

This mixed-methods study was reported in accordance with internationally recognized reporting guidelines: the TREND (Transparent Reporting of Evaluations with Nonrandomized Designs) guidelines for the quantitative quasi-experimental component, the COREQ (Consolidated Criteria for Reporting Qualitative Research) guidelines for the qualitative interview component, and the GRAMMS (Good Reporting of A Mixed Methods Study) recommendations for the overall mixed-methods design. Completed checklists for each guideline are provided in the Supplementary File S7.

## Results

### Comparison of internship scores between the two groups

The total score of the intervention group was 94.86 ± 1.28, which was higher than that of the control group (90.29 ± 1.62), and the difference between the groups was statistically significant (*t* = 17.073, *P* < 0.001). The effect size was large (d = 3.12, 95% CI: 2.53 to 3.71). In the three dimensions of theoretical performance, operational performance, and clinical internship performance, the scores of the intervention group were significantly higher than those of the control group. For details, see Table 2.

**Table 2:** Comparison of internship scores of two groups of nursing students (Mean ± SD)

Item	Intervention group (*n* = 60)	Control group (*n* = 60)	t	*P*	Cohen’s d	95% CI of d
Theoretical score	94.58 ± 1.94	90.03 ± 3.47	8.823	< 0.001	1.61	1.19, 2.03
Operational score	94.81 ± 1.35	89.48 ± 2.46	14.712	< 0.001	2.68	2.13, 3.23
Clinical practice performance	95.53 ± 1.28	92.42 ± 2.97	7.533	< 0.001	1.38	0.98, 1.78
Total scores	94.86 ± 1.28	90.29 ± 1.62	17.073	< 0.001	3.12	2.53, 3.71

### Comparison of self-directed learning ability between the two groups after teaching intervention

The total score of the intervention group was 106.35 ± 16.08, which was higher than that of the control group (99.57 ± 19.93), and the difference between the groups was statistically significant (*t* = 2.047, *P* = 0.043). The effect size was small to medium (d = 0.37, 95% CI: 0.01 to 0.73). Although there was no statistically significant difference in self-management ability between the two groups after the intervention (*P* > 0.05), the scores still showed improvement after the teaching intervention. See Table 3 for details.

**Table 3:** Comparison of self-directed learning ability of two groups of nursing students (Mean ± SD)

Item	Intervention group (*n* = 60)	Control group (*n* = 60)	t	*P*	Cohen’s d	95% CI of d
Self-management Ability	38.00 ± 5.78	39.26 ± 7.31	-1.030	0.305	-0.19	-0.55, 0.17
Information acquisition ability	41.80 ± 6.89	38.97 ± 8.09	2.031	0.045	0.37	0.01, 0.73
Learning cooperation ability	26.55 ± 4.14	24.68 ± 5.03	2.216	0.028	0.41	0.04, 0.78
Total scores	106.35 ± 16.08	99.57 ± 19.93	2.047	0.043	0.37	0.01, 0.73

### Comparison of humanistic care ability between the two groups after teaching intervention

**Fig. 3:**
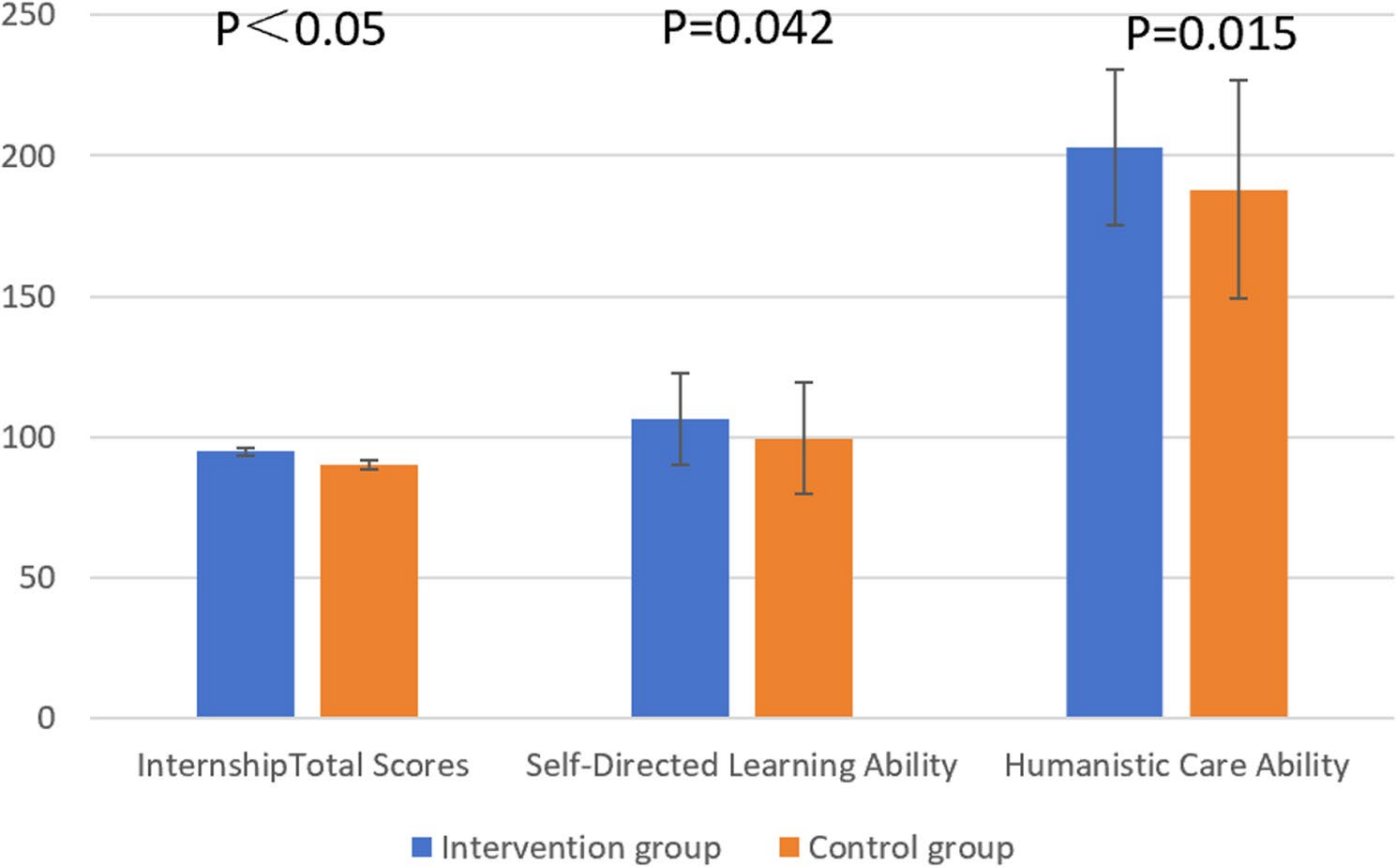
Comparison of Internship Performance, SLAS and CAI between the intervention group and control group

The total score of the intervention group was 203.08 ± 27.52, which was higher than that of the control group (187.92 ± 38.71), and the difference between the groups was statistically significant (*t* = 2.522, *P* = 0.013). The effect size was small to medium (d = 0.46, 95% CI: 0.09 to 0.82). In the three dimensions of cognition, courage, and patience, the scores of the intervention group were significantly higher than those of the control group. See Table 4 for details. The Fig. 3 shows the comparison of internship performance, SLAS and CAI between the intervention group and the control group.

**Table 4:** Comparison of Humanistic Care Ability of Two Groups of Nursing Students (Mean ± SD)

Item	Intervention group (*n* = 60)	Control group (*n* = 60)	t	*P*	Cohen’s d	95% CI of d
Cognition dimension	82.17 ± 11.03	74.13 ± 15.93	3.208	0.002	0.59	0.22, 0.96
Courage dimension	60.17 ± 18.60	59.73 ± 15.98	0.140	0.899	0.03	-0.33, 0.39
Patience dimension	60.75 ± 7.05	54.05 ± 11.71	3.800	< 0.001	0.69	0.32, 1.06
Total scores	203.08 ± 27.52	187.92 ± 38.71	2.522	0.013	0.46	0.09, 0.82

### Comparison of teaching satisfaction between the two groups

The teaching satisfaction of nursing interns in the intervention group was significantly higher than those in the control group (98.3% vs. 86.7%, χ² = 6.54, *P* = 0.038), as shown in Table 5.

**Table 5:** Comparison of Teaching Satisfaction between Two Groups of Nursing Students

Group	Vety Satisfied	Moderately Satisfied	Dissatisfied	Satisfaction Rate (%)
Intervention group (*n* = 60)	51	8	1	98.3
Control group (*n* = 60)	42	10	8	86.7
*χ²*				6.54
*P*				0.038

### Qualitative research

The qualitative data results provide information by summarizing the interview data, identifying five key themes: (a) Stimulating learning interest; (b) Enhancing self-directed learning ability; (c) Improving expressive skills and humanistic care capabilities; (d) Strengthening clinical thinking and mastery of nursing skills; (e) Suggestions for improving this teaching model. The details are as follows:

Theme 1: Stimulating learning interest.

“The typical cases presented by the teacher closely linked clinical practice with textbook knowledge, helping us better integrate theory and practice, which also motivated me to continue learning.” (S1).

“The teacher provided learning materials related to diseases before class, and the materials offered were always up-to-date. We could learn directly from them, which was quicker and more convenient than searching for information on our own.” (S2).

Theme 2: Enhancing self-directed learning ability.

“To achieve a good score on the pre-class quiz, I would instinctively review my professional textbooks first. It’s fair to say that the pre-class quiz motivated me to proactively preview the course material, while the post-class quiz helped reinforce my learning, made my review more targeted, and allowed me to retain the course content more deeply. " (S3).

“This teaching model made me more engaged in class. To avoid being asked about knowledge points from the pre-quiz that I didn’t understand, I would rush to study what I didn’t know before class. This process significantly improved my initiative in learning. " (S4).

“The virtual simulation system allowed me to identify issues in my practical operations. I would then take notes on the knowledge points I struggled with and proactively consult my instructors or search for information online. " (S5).

Theme 3: Improving expressive skills and humanistic care capabilities.

“In class, the teacher encouraged us to propose nursing measures based on the teaching cases, and everyone had the opportunity to express their opinions. During this process, I needed to concisely and effectively articulate my viewpoints, which made me consciously practice my communication skills before class.” (S6).

“Previously, my understanding of patients was limited, so my health education efforts tended to be mechanical and textbook-like. However, as the teacher guided us through targeted nursing procedures for patients in class, I felt I was stepping into the patient’s world, truly treating them as my friends. I wanted to provide better care for them, and my awareness of humanistic care also improved.” (S7).

Theme 4: Strengthening clinical thinking and mastery of nursing skills.

“Patients often decline my assistance with procedures because I am an intern. The cases designed on the virtual simulation system are highly aligned with the specific characteristics of the department, overcoming these practical barriers. They allow me to address patient issues by integrating my knowledge with my understanding, similar to a virtual experience of independently caring for patients. This has greatly honed my clinical thinking. As a result, when instructors teach nursing skills, I am more receptive and achieve better mastery.” (S8).

Theme 5: Suggestions for improving this teaching model

“While pre-class preparation and post-class review are helpful for improving my learning outcomes, they can sometimes be time-consuming and impose a slight time burden. I hope the instructor can provide more concise materials and test questions.” (S9).

“I hope the instructor can record a video explaining the questions on the virtual simulation system. This way, I can review my weak areas at any time and gain a deeper understanding of such comprehensive cases.” (S10).

Five key themes were identified from the qualitative interviews. Detailed information including subthemes, representative quotes, and frequency is provided in Supplementary File S3. To further illuminate how qualitative findings relate to the quantitative outcomes, we constructed a joint display (Supplementary File S4). The integration showed that qualitative themes consistently confirmed the quantitative improvements (e.g., internship performance), explained the mechanisms behind the effects (e.g., self-directed learning behaviors), and qualified the high satisfaction rate with concurrent suggestions for improvement.

## Discussion

According to Kirkpatrick‘s model [20] for evaluating educational outcomes, our study suggested positive effects at Level 1 (Reaction), evidenced by improved teaching satisfaction, and more importantly, at Level 2 (Learning), evidenced by enhanced theoretical knowledge, practical skills, self-directed learning abilities, and humanistic care competency. Focusing on the Level 2 outcomes assessed in our study, the findings suggested that the intervention was associated with an exceptionally large effect on internship performance (total scores: d = 3.12, 95% CI: 2.53 to 3.71). At the competency level, students achieved near mastery across all domains, which may suggest a foundation for safer, more competent bedside care, although this interpretation requires confirmation in real clinical settings. This pattern is consistent with the findings of Wei’s research [21]. Constructivist theory suggests that learning occurs when individuals actively construct their own understanding and generate new knowledge through repeated exploration, reflection, interaction, and experience within meaningful contexts [22]. Consistent with this student-centered philosophy [12], the BOPPPS model integrates pre-class preview, in-class interactive participation, and post-class review to offer students a systematic learning process [23]. Central to this approach is participatory learning, which shifts traditional “cramming teaching” toward an interactive educational model that actively involves both teachers and students [24].Based on Kolb’s experiential learning model [25], the experiences acquired by nursing students in a virtual simulation environment generate authentic insights comparable to real clinical practice. This learning approach produces more immediate and lasting outcomes, thus accounting for the effective growth of theoretical knowledge. Integrating virtual teaching technology into nursing education not only stimulates students’ learning enthusiasm and consolidates their knowledge and skills, but also lays a crucial foundation for facilitating their transition from a knowledge-oriented paradigm to a competency-driven one. Collectively, existing studies have confirmed the positive value of virtual teaching in health education. Alvitez-Temoche et al. [26] asserted that VR can create an immersive, hands-on learning environment for dental students, thereby facilitating the development of their comprehension and clinical skills. Similarly, Barteit et al. [27] demonstrated that virtual reality can also significantly enhance knowledge acquisition and skill performance among medical learners.

The results of this study showed that the total score of the self-directed learning ability in the intervention group was higher than that in the control group, indicating that the BOPPPS model with simulation system was associated with enhanced students’ self-directed learning ability. The effect size was small to moderate (total scores: d = 0.37, 95% CI: 0.01 to 0.73). Although the magnitude of this effect was modest, qualitative findings suggested potential educational value, as approximately 70% of interviewed students reported engaging in self-directed learning behaviors. The BOPPPS model may guide nursing students to think with specific questions by releasing teaching objectives and conducting pre-tests before class, thereby potentially encouraging active learning. Building on this foundation, the model uses real clinical cases as entry points, requiring students to develop individualized nursing plans for patients. This approach effectively stimulates learning interest, motivates students to actively engage in critical thinking and gather relevant knowledge, and ultimately cultivates their self-directed learning habits [28]. Additionally, this model emphasizes interactive participation among nursing students. Through diversified teaching strategies such as in-class role-playing and real-case analysis, it not only creates a positive learning environment but also enhances students’ learning enthusiasm and initiative [29]. The core concept of experiential teaching is to enable students to learn and master knowledge and skills through direct experience [30]. The virtual simulation system provides a safe, repeatable clinical experience environment in which students engage in a cyclical process of concrete experience, reflective observation, abstract conceptualization, and active experimentation. This process cultivates students’ ability to actively acquire and integrate knowledge, thereby promoting self-directed learning [31].

Caring ability is an essential competency for nursing professionals and lies at the core of nursing practice, directly influencing the quality of nursing services. Currently, the overall level of humanistic caring ability among nursing undergraduates in China is at a moderate level [32]. The results of this study demonstrated that after implementing the BOPPPS model with virtual simulation system, the intervention group scored significantly higher than the control group across all dimensions of humanistic caring ability (*P* < 0.05). The effect size was small to moderate (total score: d = 0.46, 95% CI: 0.09 to 0.82), suggesting potentially meaningful shifts in empathy orientation and patient-centered attitudes as measured by the CAI. Standardized training remains confined to the cultivation of theoretical knowledge and practical skills, resulting in a notable lack of soft skills training for nursing students. In contrast, the BOPPPS model emphasizes not only the application of clinical expertise and technical skills but also the development of individual non-technical abilities [33]. During the participatory learning phase of the new teaching model, targeted scenario-based simulation training was implemented, allowing nursing students to engage in role-playing from the patient’s perspective. This approach enabled them to learn how to express caring toward patients and others, enhanced their understanding of humanistic concepts, and cultivated their humanistic caring ability [34]. Furthermore, the BOPPPS model with virtual simulation system may help create diverse simulated clinical scenarios tailored to specific teaching objectives and content. Through this immersive, clinic-like environment, nursing students can deepen their comprehension and internalization of knowledge while simultaneously experiencing the importance of humanistic caring.

Beyond objective learning outcomes, teaching satisfaction is another important dimension of educational evaluation. Student satisfaction with teaching is typically defined as the proportion or percentage of students who express overall satisfaction with the teaching process, reflecting their level of approval and satisfaction with the educational program [35]. The BOPPPS model is grounded in the core concept of equality between teachers and students, explicitly positioning students as the primary drivers of the learning process [36]. By employing diversified classroom interactive activities—including both student-student and teacher-student interactions—this model breaks away from traditional teaching paradigms, facilitates students’ transition from passive reception to active inquiry, and thereby constructs a stimulating and dynamic learning environment [37]. This atmosphere enhances students’ motivation to participate in educational activities, promotes the development of their comprehensive skills, and improves their learning interest and efficacy. Students generally prefer interactive and engaging courses, which make them more willing to actively engage in teaching activities, thereby increasing their satisfaction with teaching quality. The virtual simulation system provides a robust technical environment for collaborative learning and effectively promotes teacher-student interaction. Through group discussions and collaborative activities in virtual reality, learners deepen their problem-solving and critical thinking abilities, enhance their cognitive skills, and simultaneously strengthen their collaborative communication competence [38]. This gradual improvement in collaborative communication skills, in turn, increases nursing students’ academic satisfaction.

However, the study has certain limitations: Firstly, the sample size was relatively small, the single-center design limits generalizability, and the short intervention duration (4 weeks) precludes assessment of long-term outcomes. Furthermore, differences persist between virtual simulation system and real-world clinical scenarios, and the long-term impact of this model on nursing students’ clinical adaptability warrants further longitudinal evaluation. Future randomized controlled trials with multi-center designs and longer follow-up are needed to confirm our findings. Secondly, this study used single item exploratory indicators to capture students’ perceived changes in professional identity and humanistic care awareness. Future studies should employ validated, multi-item scales to more rigorously evaluate these outcomes. Thirdly, the non-randomized, temporally separated design (control group in 2023, intervention group in 2024 to 2025) may introduce selection bias, historical effects, cohort effects, andunmeasured temporal confounders, which limits causal inference. Additionally, the Hawthorne effect and social desirability bias in self-report measures cannot be excluded. Fourthly, teaching satisfaction was measured using a self-developed unidimensional scale with limited response granularity. This exploratory measure does not capture multidimensional aspects of satisfaction (e.g., course content, instructor effectiveness, technical usability). Future studies should employ validated, multi-dimension satisfaction scales. Finally, this study did not evaluate cost-effectiveness, which is important for informing resource allocation decisions.

### Practical implications

The BOPPPS model with virtual simulation system entails low costs and minimal resource demands, as it leverages existing simulation platforms and basic hardware. Brief instructor training ensures consistent delivery, and the model integrates seamlessly into current clinical internship curricula. It is also scalable to other surgical nursing specialties. Potential barriers, such as limited access to technology, faculty resistance, and time constraints, can be effectively addressed through institutional support and streamlined teaching materials. Aligned with contemporary nursing education standards, the model may inform policy recommendations aimed at expanding blended, simulation-based training nationwide.

## Conclusions

The BOPPPS model with virtual simulation system shows promising associations with improved objective learning outcomes among nursing interns in the otolaryngology-head and neck surgery department, including theoretical knowledge, practical skills, self-directed learning abilities, and humanistic care competency. In addition, students’ satisfaction with the teaching approach was also improved. Qualitative interviews further identified positive themes, including heightened learning motivation, increased autonomy, improved communication and empathy skills, and enhanced clinical thinking and skill mastery. These findings reflect the multidimensional value of this integrated model in promoting the holistic development of nursing students.

## Supplementary Information

Supplementary Material 1.

Supplementary Material 2.

Supplementary Material 3.

Supplementary Material 4.

Supplementary Material 5.

Supplementary Material 6.

Supplementary Material 7.

## Data Availability

The datasets generated and/or analyzed during the current study are available from the corresponding author on reasonable request.
