# Supplementary material for: BOPPPS model with virtual simulation system for otorhinolaryngology head and neck surgery nursing interns: a quasi-experimental study
Source: BMC Med Educ. 2026 Jun 8;26:1110. doi: 10.1186/s12909-026-09648-z (PMC13348939; doi:10.1186/s12909-026-09648-z)
Supplement: Supplementary file 1 — Supplementary Material 1. [file 12909_2026_9648_MOESM1_ESM.docx]

|  |  |
| --- | --- |
|  |  |
|  |  |
|  |  |
|  |  |

**Table S1.** Psychometric properties of the teaching satisfaction scale

| Verification indicators | Result | criteria |
| --- | --- | --- |
| Content Validity (I-CVI) | 0.80 to 1.00 | ≥0.78 acceptable |
| Content Validity (S-CVI/Ave) | 0.92 | ≥0.90 excellent |
| Internal consistency (Cronbach‘s α) | 0.870 | >0.70 acceptable |
| EFA variance explained | 68.4% | >50% acceptable |
