# Supplementary material for: BOPPPS model with virtual simulation system for otorhinolaryngology head and neck surgery nursing interns: a quasi-experimental study
Source: BMC Med Educ. 2026 Jun 8;26:1110. doi: 10.1186/s12909-026-09648-z (PMC13348939; doi:10.1186/s12909-026-09648-z)
Supplement: Supplementary file 2 — Supplementary Material 2. [file 12909_2026_9648_MOESM2_ESM.docx]

**Appendix S1.** Semi-Structured Interview Guide

**Opening script:** “Thank you for participating in this interview. This interview aims to understand your experience with the BOPPPS model with virtual simulation system. Your responses will be kept confidential and will not affect your grades. The interview will take about 15 to 20 minutes.”

**Core questions:**

1.What are your overall thoughts on the blended teaching approach (BOPPPS model with virtual simulation system) in otorhinolaryngology head and neck surgery?

2.How has this teaching model affected your learning outcomes compared to traditional methods? (Probe: theoretical knowledge, practical skills, and self-directed learning)

3.How has it affected your communication skills and humanistic care awareness?

4.How has it influenced your clinical thinking and mastery of nursing skills?

5.What suggestions do you have for improving this teaching model?

6.Is there anything else you would like to share?

**Closing script:** “Thank you for your time and valuable feedback.”
