# Supplementary material for: BOPPPS model with virtual simulation system for otorhinolaryngology head and neck surgery nursing interns: a quasi-experimental study
Source: BMC Med Educ. 2026 Jun 8;26:1110. doi: 10.1186/s12909-026-09648-z (PMC13348939; doi:10.1186/s12909-026-09648-z)
Supplement: Supplementary file 3 — Supplementary Material 3. [file 12909_2026_9648_MOESM3_ESM.docx]

**Table S1.** Qualitative Themes with Subthemes, Representative Quotes, and Frequency (n=14)

| Theme | Subtheme | Frequency (n) | Representative Quote |
| --- | --- | --- | --- |
| 1. Stimulating learning interest | — | 12 | “The typical cases presented by the teacher closely linked clinical practice with textbook knowledge, helping us better integrate theory and practice, which also motivated me to continue learning.” (S1) |
| 2. Enhancing self-directed learning ability | Pre-class preview | 10 | “To achieve a good score on the pre-class quiz, I would instinctively review my professional textbooks first. The pre-class quiz motivated me to proactively preview the course material.” (S3) |
|  | Post-class review | 8 | “The post-class quiz helped reinforce my learning, made my review more targeted, and allowed me to retain the course content more deeply.” (S4) |
|  | Independent problem-solving | 7 | “The virtual simulation system allowed me to identify issues in my practical operations. I would then take notes on the knowledge points I struggled with and proactively consult my instructors or search for information online.” (S5) |
| 3. Improving expressive skills and humanistic care capabilities | Communication skills | 9 | “In class, the teacher encouraged us to propose nursing measures based on the teaching cases, and everyone had the opportunity to express their opinions. During this process, I needed to concisely and effectively articulate my viewpoints, which made me consciously practice my communication skills before class.” (S6) |
|  | Humanistic care awareness | 11 | “As the teacher guided us through targeted nursing procedures for patients in class, I felt I was stepping into the patient's world, truly treating them as my friends. I wanted to provide better care for them, and my awareness of humanistic care also improved.” (S7) |
| 4. Strengthening clinical thinking and mastery of nursing skills | Clinical thinking | 10 | “The cases designed on the virtual simulation system are highly aligned with the specific characteristics of the department. They allow me to address patient issues by integrating my knowledge with my understanding, similar to a virtual experience of independently caring for patients. This has greatly honed my clinical thinking.” (S8) |
|  | Skill mastery | 9 | “When instructors teach nursing skills, I am more receptive and achieve better mastery.” (S9) |
| 5. Suggestions for improving this teaching model | Concise learning materials | 6 | “While pre-class preparation and post-class review are helpful for improving my learning outcomes, they can sometimes be time-consuming and impose a slight time burden. I hope the instructor can provide more concise materials and test questions.” (S10) |
|  | Video explanation of simulation questions | 5 | “I hope the instructor can record a video explaining the questions on the virtual simulation system. This way, I can review my weak areas at any time and gain a deeper understanding of such comprehensive cases.” (S11) |
