# Supplementary material for: BOPPPS model with virtual simulation system for otorhinolaryngology head and neck surgery nursing interns: a quasi-experimental study
Source: BMC Med Educ. 2026 Jun 8;26:1110. doi: 10.1186/s12909-026-09648-z (PMC13348939; doi:10.1186/s12909-026-09648-z)
Supplement: Supplementary file 4 — Supplementary Material 4. [file 12909_2026_9648_MOESM4_ESM.docx]

**Table S1.** Joint Display of Quantitative and Qualitative Findings

| Quantitative Outcome | Quantitative Result  (Intervention vs. Control) | Qualitative Finding | Integration & Interpretation |
| --- | --- | --- | --- |
| Internship performance (total scores) | 94.86±1.28 vs. 90.29±1.62 *t*=17.073, *P*<0.05 d=3.12 | Theme 1: “The typical cases helped us better integrate theory and practice, which motivated me to continue learning.” (S1) Theme 4: “The system greatly honed my clinical thinking.” (S8) | **Qualitative findings explain the “how”:** Students attributed their improved performance to the integration of theory and practice (Theme 1) and enhanced clinical thinking (Theme 4). |
| Self-directed learning ability (total scores) | 106.35±16.08 vs. 99.57±19.93 *t*=2.047, *P=*0.043 d=0.37 | Theme 2: “The pre-class quiz motivated me to proactively preview... the post-class quiz helped reinforce my learning.” (S3) “The virtual simulation system allowed me to identify issues... I would proactively consult instructors or search for information online.” (S5) | **Qualitative findings reveal mechanisms:** Students described specific behaviors driving self-directed learning (pre-class preview, post-class review, independent problem-solving). |
| Humanistic care ability (total scores) | 203.08±27.52 vs. 187.92±38.71 *t=*2.522, *P=*0.013 d=0.46 | Theme 3: “I felt I was stepping into the patient’s world, truly treating them as my friends. I wanted to provide better care for them.” (S7) “I needed to concisely articulate my viewpoints... my awareness of humanistic care also improved.” (S6) | **Qualitative findings add depth:** Students described an emotional shift (empathy, patient-centeredness) that quantitative scores alone cannot capture. |
| Teaching satisfaction | 98.3% vs. 86.7% χ²=6.54, *P*=0.038 | Theme 5 (suggestions): “Pre-class preparation can be time-consuming... I hope the instructor can provide more concise materials.” (S10) | **Qualitative findings qualify satisfaction:** While satisfaction was high (98.3%), students also identified areas for improvement (e.g., concise materials, video explanations). This suggests that satisfaction is not absolute but balanced with perceived burden. |

**Figure A.** Map of Qualitative Findings
