# Supplementary material for: BOPPPS model with virtual simulation system for otorhinolaryngology head and neck surgery nursing interns: a quasi-experimental study
Source: BMC Med Educ. 2026 Jun 8;26:1110. doi: 10.1186/s12909-026-09648-z (PMC13348939; doi:10.1186/s12909-026-09648-z)
Supplement: Supplementary file 5 — Supplementary Material 5. [file 12909_2026_9648_MOESM5_ESM.docx]

Theme 2:

Self-directed learning ability

Theme 3:

Expressive skills & humanistic

care capabilities

Theme 4:

Clinical thinking & skill mastery apabilities

**Figure A.** Map of Qualitative Findings

Theme 5:

Suggestions for improvement

Enhanced Learning Outcomes

Theme 1: Stimulating learning interest

BOPPPS model with virtual simulation system
