# Supplementary material for: BOPPPS model with virtual simulation system for otorhinolaryngology head and neck surgery nursing interns: a quasi-experimental study
Source: BMC Med Educ. 2026 Jun 8;26:1110. doi: 10.1186/s12909-026-09648-z (PMC13348939; doi:10.1186/s12909-026-09648-z)
Supplement: Supplementary file 6 — Supplementary Material 6. [file 12909_2026_9648_MOESM6_ESM.docx]

Intervention Protocol

**Appendix A. Intervention Overview**

| **Item** | **Description** |
| --- | --- |
| Duration | 4 weeks, 1 session/week, 60 min/session |
| Platform | National Virtual Simulation Experiment Teaching Course Sharing Platform |
| Course | airway suction in adults undergoing invasive mechanical ventilatio |
| Hardware | VR headsets (HTC Vive) or touchscreens |

**Appendix B.** Lesson Plan

| **BOPPPS Phase** | **Activity** | **Time** |
| --- | --- | --- |
| Bridge-in | Video case (post‑laryngectomy patient) + 3 guided questions | 5 min |
| Objective | State 3 learning objectives (knowledge, skill, emotional) | 3 min |
| Pre-assessment | Online quiz + VR demonstration with real‑time feedback | 7 min |
| Participatory learning | Group VR simulation + role‑play + team emergency response | 30min |
| Post-assessment | System scoring + blinded head nurse grading | 10 min |
| Summary | Error analysis + mind map + homework | 5 min |

**Appendix C.** Simulation Scenarios with Learning Objectives

| **Scenario** | **Learning Objectives** |
| --- | --- |
| Wound bleeding | Recognize bleeding signs, prioritize actions, communicate with non‑verbal patient |
| Tracheostomy suctioning | Prepare equipment, perform sterile suctioning, monitor patient response |
| Emergency airway obstruction | Recognize obstruction, perform emergency suctioning, call for assistance |
| Discharge education | Teach home tracheostomy care, provide speech rehabilitation resources |

**Appendix D.** Assessment Checklist (Suctioning)

| **Step** | **Task** | **Pass** |
| --- | --- | --- |
| 1 | Verify order and explain procedure to patient | 🞎 |
| 2 | Set suction pressure (80 to 120 mmHg) | 🞎 |
| 3 | Select appropriate catheter (12 to 14 Fr) | 🞎 |
| 4 | Perform hand hygiene and maintain sterile technique | 🞎 |
| 5 | Pre‑oxygenate patient | 🞎 |
| 6 | Insert catheter without suction | 🞎 |
| 7 | Apply suction while withdrawing (≤15 seconds) | 🞎 |
| 8 | Monitor oxygen saturation | 🞎 |
| 9 | Limit passes to 2 to 3 | 🞎 |
| 10 | Document procedure | 🞎 |

Note: Passing score: ≥8/10 (80%)
