# Supplementary material for: BOPPPS model with virtual simulation system for otorhinolaryngology head and neck surgery nursing interns: a quasi-experimental study
Source: BMC Med Educ. 2026 Jun 8;26:1110. doi: 10.1186/s12909-026-09648-z (PMC13348939; doi:10.1186/s12909-026-09648-z)
Supplement: Supplementary file 7 — Supplementary Material 7. [file 12909_2026_9648_MOESM7_ESM.docx]

**Table S1.** TREND Checklist for Non-Randomized Designs

| Section | Item | Description | Reported in manuscript |
| --- | --- | --- | --- |
| Title and abstract | 1 | Information on how units were allocated (e.g., quasi‑experimental) | Title, Abstract |
| Background | 2 | Scientific background and rationale | Introduction |
| Participants | 3 | Eligibility criteria, settings, data collection methods | 2.1. Participants |
| Intervention | 4 | Detailed description of intervention and control conditions | 2.2. Teaching methods |
| Objectives | 5 | Specific objectives and hypotheses | 1.Introduction |
| Outcomes | 6 | Clearly defined primary and secondary outcomes | 2.3.Teaching effectiveness evaluation |
| Sample size | 7 | How sample size was determined | 2.1. Participants |
| Assignment method | 8 | Unit of assignment (e.g., temporal separation) | 2.1. Participants |
| Blinding | 9 | Whether participants, instructors, or assessors were blinded | 2.2. Teaching methods |
| Baseline data | 10 | Baseline characteristics by group | 2.1. Participants,Table 1 |
| Statistical methods | 11 | Statistical methods used to compare groups | 2.4. Statistical analysis |
| Outcomes and estimation | 12 | For each outcome, effect size and precision | 3.Results, Tables 2 to Table 5 |
| Ancillary analyses | 13 | Any other analyses performed (e.g., ANCOVA sensitivity) | Not applicable |
| Adverse events | 14 | Any unintended effects | None occurred |
| Interpretation | 15 | Interpretation consistent with results | 4.Discussion |
| Generalizability | 16 | External validity | Limitations |

Note: TREND, Transparent Reporting of Evaluations with Nonrandomized Designs.

**Table S2.** COREQ Checklist for Qualitative Research

| Item | Description | Reported in manuscript |
| --- | --- | --- |
| **Domain 1: Research team and reflexivity** |  |  |
| 1. Interviewer/facilitator credentials | Research team qualifications | Authors’ affiliations |
| 2. Relationship with participants | No prior relationship | 2.2. Teaching methods |
| 3. Participant knowledge of researcher | Knew interviewer was a researcher | 2.2. Teaching methods |
| **Domain 2: Study design** |  |  |
| 4. Sampling strategy | Purposive sampling | 2.1. Participants |
| 5. Setting for data collection | Hospital classroom | 2.3.5.Qualitative evaluation - semistructured interviews |
| 6. Presence of non‑participants | Only interviewer and participant | - |
| 7. Interview guide | Three core questions | 2.3.5.Qualitative evaluation - semistructured interviews |
| 8. Repeat interviews | No | - |
| 9. Audio/visual recording | Audio recording | - |
| 10. Field notes | Yes | - |
| 11. Duration | 15‑20 minutes | 2.3.5.Qualitative evaluation - semistructured interviews |
| 12. Data saturation | Reached after 12 interviews | 2.3.5.Qualitative evaluation - semistructured interviews |
| **Domain 3: Analysis and findings** |  |  |
| 13. Number of coders | Two independent coders | 2.3.5.Qualitative evaluation - semistructured interviews |
| 14. Coding tree description | Provided (themes with subthemes) | Supplementary File S3 |
| 15. Derivation of themes | Inductive thematic analysis | 2.3.5.Qualitative evaluation - semistructured interviews |
| 16. Software used | NVivo 14 | 2.3.5.Qualitative evaluation - semistructured interviews |
| 17. Participant checking | Member checking performed | 2.3.5.Qualitative evaluation - semistructured interviews |
| 18. Quotations presented | Yes, anonymized | 2.3.5.Qualitative evaluation - semistructured interviews |
| 19. Consistency with findings | Themes supported by quotations | Supplementary File S3 |
| 20. Clarity of themes | Clearly reported | Supplementary File S3 |

Note: COREQ, Consolidated Criteria for Reporting Qualitative Research.

**Table S3. GRAMMS Checklist for Mixed-Methods Studies**

| Item | Description | Reported in manuscript |
| --- | --- | --- |
| 1 | Describe the justification for using mixed methods | Abstract (mixed‑methods approach) |
| 2 | Describe the design (e.g., sequential, concurrent, priority) | 2. Methods |
| 3 | Describe each method in sufficient detail | Quantitative (2.2.2.2. Teaching implementation);  Qualitative (2.3.5. Qualitative evaluation - semistructured interviews) |
| 4 | Describe where integration occurred | Supplementary File S4 |
| 5 | Describe any limitations of mixing methods | Discussion (Limitations) |
| 6 | Describe insights gained from mixing methods | Discussion |

Note: GRAMMS, Good Reporting of A Mixed Methods Study.
